# Investigation study data to develop sustainable concrete mix using waste materials as constituents

**DOI:** 10.1016/j.dib.2023.109837

**Published:** 2023-11-20

**Authors:** Nelson Ponnu Durai T., Kandasamy S.

**Affiliations:** Vel Tech Rangarajan Dr. Sagunthala R&D Institute of Science and Technology, Avadi, Chennai, 600062, Tamil Nadu, India

**Keywords:** Sustainable Concrete, GGBFS, Metakolin, Alccofine, Copper Slag, Foundry Sand, Sintered Fly ash aggregates, Recycled aggregates

## Abstract

Sustainable construction materials are those which contributes towards the carbon- negative process of manufacturing. Cement produced by raw materials from industrial wastes and non-fossil fuel sources is considered as green cement which has high potential in constructions due to high tensile strength and resistance to corrosion. Exploring replacement materials for conventional cement is an active area of research. This data investigation focused on development of novel concrete mix with various proportions of sustainable supplementary materials with cement, fine and coarse aggregate substances. Alccofine, Metakaolin, GGBFS, Foundry sand, Copper slag, Recycled aggregate and Sintered fly ash aggregate are suitable supplementary materials of the concrete mix. Data projected replacement of cement by 15 % Alccofine, 10% Metakaolin and 30% GGBFS substitution and Fine aggregates (50% Copper slag, 30% Foundry sand) and replacement of Coarse aggregate (20% Recycled and 30% Sintered Fly Ash aggregate) will produce sustainable concrete mixture. Compressive and split tensile strength examined at 7^th^, 14^th^ and 28^th^ day and compared with conventional concrete. This data shows that the concrete mixture CFACA 1234567 was outperformed among the five mixture studied.

## Graphical abstract

**Figure:**
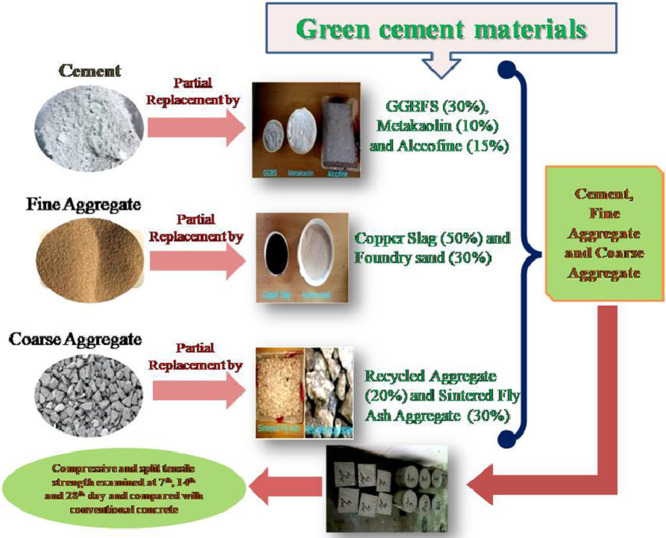

Specifications Table

**Table:** 

Subject	Civil Engineering
Specific subject area	Sustainable Concrete*,* supplementary constituents
Data format	Raw and Analyzed
Type of data	Text, Table and Figure
Data collection	To prepare the data, we collected waste Cementitious materials like GGBFS, Metakaolin and Alccofine were used as a replacement for cement at a Percentage of optimum 30, 10 and 15. Copper slag and foundry sand were used as substitutes for fine aggregate at a Percentage of optimum 50 and 30. Sintered fly ash aggregate and recycled aggregate as an alternative for coarse aggregate at a Percentage of optimum 30 and 20.
Data source location	• Institution: Vel Tech Rangarajan Dr. Sagunthala R&D Institute of Science and Technology • City/Town/Region: Chennai, Tamil Nadu • Country: India
Data accessibility	Repository name: Mendeley Data Data identification number: 10.17632/p2w6rf3gs7.1 Direct URL to data: https://data.mendeley.com/datasets/p2w6rf3gs7/1

## 1 Value of the Data

• These data are valuable because they represent a comprehensive approach to producing sustainable concrete mixtures. The combination of cement replacement materials like Alccofine, Metakaolin, and GGBFS, along with the use of alternative materials for both fine and coarse aggregates, demonstrates a commitment to reducing the environmental impact of concrete production. By reducing the use of Portland cement, which is a major source of carbon emissions, and incorporating industrial by-products like copper slag, foundry sand, recycled aggregate, and sintered fly ash aggregate, this approach not only conserves natural resources but also minimizes waste. Sustainable concrete mixtures like this align with global efforts to mitigate climate change and promote eco-friendly construction practices, making them highly valuable for sustainable infrastructure development.
• 8% of the world's CO_2_ emissions are due to cement manufacturing. In addition, the extraction of raw materials for concrete, including sand and gravel, results in the damage of the environment and water contamination. Numerous benefits are available for sustainable concrete in the areas of the economy, society, and the environment. These advantages add up to a more sustainable and accountable building business. Contractors, builders, and construction companies can use these data to develop sustainable concrete mixtures, reducing their reliance on traditional cement and natural aggregates, and aligning with environmental regulations and sustainable construction practices. For instance, it is noteworthy that a sustainable option with less environmental impact is advantageous over the traditional 1 cubic meter concrete mixer, which contains 394 kg of cement, 743 kg of fine aggregate and 1115 kg of coarse aggregate. One can achieve resource efficiency while also improving the environmental impact by replacing 15 % of cement with Alccofine, 10 % with Metakaolin and 30 % with GGBFS, along with 50 % Copper slag and 30 % Foundry sand in place of fine aggregates, and 20 % Recycled aggregates and 30 % Sintered fly ash aggregate for coarse aggregate. Hence, the composition changes to 177.3 kg of cement, 148.6 kg of fine aggregate and 557.5 kg of coarse aggregate which is now being used in this study, the altered combination greatly reduce carbon emissions related to cement manufacture and lessens its ecological impact from the construction.
• A number of researchers have recently been working on developing sustainable concrete for commercial usage incorporating recycled materials. Recycling construction materials is of the utmost importance because of the growing changing global environment. The intention of the present investigation is to develop sustainable concrete for application in construction projects in the future while also addressing the issue of recycling materials. For recycled concrete to be strong and high-quality, several recycled elements must be used. The current research includes a knowledge of and application of various components in concrete mixtures. Researchers can analyse the cost implications of adopting these sustainable concrete mixtures, considering factors such as material costs, transportation, and long-term maintenance. Understanding the economic benefits can encourage wider adoption in the construction industry.

## 2 Objective

To explore and evaluate sustainable practices and materials in concrete production with a focus on the utilization of supplemental cementitious materials (SCMs) such as ground granulated blast-furnace slag (GGBFS), Alccofine, and metakaolin, as well as the incorporation of copper slag, recycled concrete aggregate (RCA), and sintered fly ash aggregates. Additionally, the objective is to assess their environmental benefits, strength development characteristics, and their potential to mitigate the depletion of natural resources, with the aim of promoting more eco-friendly and resource-efficient construction practices.

## 3 Data Description

The recent studies on replacement of ingredients of conventional concrete are having the limitations on their scope of replacement in any of the three main ingredients namely, cement, fine aggregates and coarse aggregates by using the corresponding materials which possess similar properties of the replacing ingredients. In this study, it is aimed to simultaneously replace all the ingredients of conventional concrete by the most promising materials at individual replacement studies. But it is important to note that, when the replacement is simultaneous, the percentage of addition of replacing materials cannot be taken as the percentage of optimum replacement in the individual replacement studies. Hence, the problem lies in identifying the optimum percentage of replacement of all the materials when they are used for simultaneous replacement. The data has been prepared by taking this constraint in consideration and tests have been conducted with the marginal values on either side from the individual optimum value.

The dataset collected and described in this investigation study provides insights into the development and assessment of sustainable concrete mixtures using various supplementary materials. The data analysis serves to validate the potential benefits of such sustainable mix designs in terms of strength and environmental impact, offering valuable insights for the construction industry's transition towards more eco-friendly and high-performance concrete solutions.

File 1, a supplementary information, shows the flakiness and elongation index test results for different groups of sizes of recycled aggregates used in the study, as per IS 2386 Part 1:1963. In general, the flakiness index and elongation index should not exceed 30% and 45% respectively and the corresponding results 6.08% and 20.9% indicate that both the flakiness and elongation indices are within the usable range. The summary of the indices are given here in Table 1.

**Table 1:** Flakiness Index and Elongation Index.

S.No.	Tests	Recycled Coarse Aggregate Material
1.	Flakiness Index	6.08%
2.	Elongation Index	20.90%

File 2, a supplementary information, shows the aggregates impact strength of conventional natural aggregate recycled and sintered fly ash aggregates used in the study, respectively, in row wise. The columns consist of calculation of impact strength test. Test results of natural aggregate, recycled aggregates and sintered fly ash aggregates are 6.25, 15 and 26.32 respectively. These results assist in selecting the most appropriate aggregate type for specific construction or engineering needs, with numerical values providing insight into their unique characteristics and suitability for different applications. The abstract of the values of impact strength is given in Table 2.

**Table 2:** Impact strength.

S.No.	Tests	Materials		
		Coarse Aggregate	Recycled Coarse Aggregate	Sintered Fly Ash Aggregate
1.	Impact Test	I=6.25%	I=15%	I=26.32%

File 3, a supplementary information, shows the sieve analysis data of sieve analysis data segregated the different types of aggregates namely, conventional Fine aggregates, Foundry sand, Copper slag, Coarse aggregate, Recycled aggregates in row wise. Each aggregate is sieved as per Indian Standard sieves and under each type of aggregates the sieve sizes are mentioned against the serial no. The columns consist of the remaining data such as, sieve size, mass of sample retained in each sieve, percentage of mass retained and so on for the calculation fineness modulus of the aggregate as per the standard procedure. The Table 3 contains the abstract of the results of fineness modulus from the sieve analysis for the conventional fine aggregates, foundry sand, copper slag, coarse aggregate, recycled aggregates and sintered fly ash. In general the fineness modulus for the fine aggregates should not be less than 1.4 and 1.5 in case of crushed sand and gravels; and natural sand.  The results indicate that for all the fine aggregates i.e., conventional fine aggregates, foundry sand, copper slag and sintered fly ash are having fineness modulus above the reference values. For the coarse aggregates the value should be within the range between 6 and 10. But the recycled aggregate here shows 4.16 as fineness modulus which indicates it is mixed with some quantity of fine aggregates and in recycled aggregates this is acceptable.

**Table 3:** Properties of Supplementary Constituents - Sieve Analysis.

Sl.No.	Test Name	Materials	Fineness Modulus
1.	Sieve Analysis (Fineness Modulus)	Fine Aggregate	3.12
2.		Foundry Sand	1.96
3.		Copper Slag	3.08
4.		Coarse Aggregate	4.35
5.		Recycled Coarse Aggregate	4.16
6.		Sintered Fly Ash Aggregate	2.24

File 4, a supplementary information, shows the specific gravity for the cement, alccofine, metakaolin,  GGBFS, conventional fine aggregate, coarse aggregate, sintered fly ash and recycled aggregate in row wise data. In column wise, the data required to calculate the specific gravity are given and the final values are reproduced in Table 4. This data is important to achieve the proper mix proportions.

**Table 4:** Properties of Supplementary Constituents–Specific Gravity.

Sl.No.	Test Name	Materials	Specific Gravity
1.	Specific Gravity	Cement	2.56
2.		Alccofine	1.67
3.		Metakaolin	3.71
4.		GGBFS	2.2
5.		Fine Aggregate	1.79
6.		Coarse Aggregate	2.8
7.		Sintered Fly Ash Aggregate	2.03
8.		Recycled Coarse Aggregate	2.36

File 5, in the dataset shows the compressive and split tensile strengths analysis. In compressive strength analysis data, the compressive strength for the conventional concrete, concrete with replacement of cement, replacement of fine aggregate, replacement of coarse aggregate and replacement of all the conventional concrete ingredients have been presented row wise. For each type of concrete three sets of specimens have been prepared and tested as per IS guidelines, hence it forms 15 rows.

Similarly, for the splitting tensile strength 15 rows of data have been produced. In the columns, the data of load taken by the samples and the corresponding strengths are given in three columns as the strengths have been tested on days 7, 14 and 28. The results indicate that of all types of concrete made by the replacement of either cement or fine aggregate or coarse aggregate or all the ingredients altogether, both the compressive as well as splitting tensile strengths are found to be greater than the conventional concrete.

## 4 Experimental Design, Materials and Methods

**Fig. 4.1:**
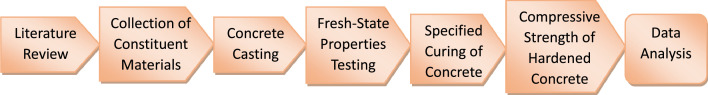
Flow Chart of research design and methodology.

The Fig. 4.1 offers a clear and concise overview of how data was collected, analyzed, and interpreted in the study, making it a valuable reference point for readers seeking to understand the research design and methodology.

### 4.1 Constituent Material

#### 4.1.1 Cement

Cement is the binding agent that holds the concrete mixture together. It is typically made from a combination of limestone, clay, shells, silica, and iron ore. Portland cement is the most common type used in concrete production. It reacts with water to form a solid matrix that binds the other materials together.

#### 4.1.2 Aggregates

Aggregates make up the bulk of concrete's volume. They include both fine and coarse materials:

• Fine Aggregate: Usually sand or crushed stone, fine aggregate provides cohesion to the concrete mix and fills the spaces between coarse aggregate particles.
• Coarse Aggregate: Typically crushed stone, gravel, or recycled concrete, coarse aggregate adds strength and stability to the concrete mixture.

#### 4.1.3 Water

Water is essential for the hydration process of cement. It reacts with the cement particles to form the solid structure of concrete. Proper control of water content is critical to achieving the desired properties of the concrete, including strength and workability.

#### 4.1.4 Supplementary Materials

Incorporating supplementary cementitious materials (SCMs) into concrete mixtures can significantly enhance various properties of concrete while also promoting sustainability. In the composition provided, which includes 15% Alccofine, 10% Metakaolin, and 30% GGBFS (Ground Granulated Blast Furnace Slag), along with specific replacements for fine aggregates, here are the key points regarding these SCMs:

1. Alccofine (15%): Alccofine is a high-performance microfine supplementary cementitious material. When used in concrete, it contributes to improved workability, increased durability, and enhanced strength development. Its fine particle size allows it to fill in gaps between larger particles, leading to a denser and more impermeable concrete matrix [[1], [2]].
2. Metakaolin (10%): Metakaolin is a pozzolan, which means it reacts with calcium hydroxide in the presence of water to form additional binding materials (calcium silicate hydrates and calcium aluminate hydrates). The inclusion of metakaolin can enhance the early-age strength and reduce the susceptibility of concrete to cracking while also improving resistance to chemical attacks [[3], [4]].
3. GGBFS (30%): Ground Granulated Blast Furnace Slag is a byproduct of the iron and steel industry. GGBFS is known for its excellent pozzolanic and latent hydraulic properties. When incorporated into concrete, it can enhance long-term strength, reduce heat of hydration, and improve resistance to sulfate and chloride attacks. It also contributes to sustainability by utilizing industrial waste [[5], [6]].

In addition to these SCMs, the composition also specifies replacements for fine and coarse aggregates:

1. Copper Slag (50%): Replacing 50% of the fine aggregate with copper slag not only reduces the demand for natural sand but also offers advantages like improved workability, increased durability, and potential cost savings. Copper slag is a byproduct of the copper extraction process [[7], [8]].
2. Foundry Sand (30%): Replacing 30% of the fine aggregate with foundry sand, another industrial byproduct, can contribute to sustainable construction practices. It may influence properties like workability and compressive strength depending on its particle size and characteristics [[9], [10]].
3. Recycled Aggregate (20%): The inclusion of 20% recycled aggregate promotes recycling and sustainability in construction. However, the properties of recycled aggregates can vary, which may affect concrete characteristics. Proper quality control is crucial when using recycled aggregates [[11], [12]].
4. Sintered Fly Ash Aggregate (30%): Sintered Fly Ash aggregate is created by sintering fly ash particles. Its use can improve the environmental profile of concrete by reducing the demand for natural aggregates. It may also offer enhanced durability properties, depending on the manufacturing process [[13], [14]].

In summary, the use of these supplementary cementitious materials and alternative fine aggregates in the concrete mixture represents a sustainable approach that aims to improve concrete performance while minimizing environmental impact through the utilization of industrial byproducts and recycled materials.

### 4.2 Mix Proportioning

The most frequently used produced building material worldwide is regarded as being concrete. The majority of the time, cement, coarse aggregate, fine aggregate, admixtures, water, etc. are mixed to create it. The compressive strength and split tensile strength test values compared to ordinary concrete mix indicate improved results when cement, sand, and coarse particles are changed. In this investigation, various supplementary constituents were used to identify the replacements for Cement, Fine aggregate, Coarse aggregate.

The Concrete Specimen are casted in M20 Grade as per IS 1026-2019 [15]**.** The various supplementary constituents for cement, fine aggregate and coarse aggregates. Cementitious materials like GGBFS, Metakaolin and Alccofine were used as a replacement for cement at a Percentage of optimum 30, 10 and 15. Copper slag and foundry sand were used as substituents for fine aggregate at a Percentage of optimum 50 and 30. Sintered fly ash aggregate and recycled aggregate as an alternative for coarse aggregate at a Percentage of optimum 30 and 20. Different Mix Proportion are detailed below in Table 5.

**Table 5:** Mix Proportion.

Mix Details	Cement (Kg/m^3^)	Fine aggregate (Kg/m^3^)	Coarse aggregate (Kg/m^3^)	Water (Kg/m^3^)	GGBFS (30%)	Metakaolin (10%)	Alccofine (15%)	Copper Slag (50%)	Foundry Sand (30%)	Sintered fly ash aggregate (30%)	Recycled Aggregate (20%)
Control Concrete	411	719	1327	197	-	-	-	-	-	-	-
C123	184	719	1327	197	123	41	62	-	-	-	-
FA 45	411	144	13274	197	-	-	-	359	216	-	-
CA 67	411	719	664	197	-	-	-	-	-	398	265
CFACA 1234567	184	144	664	197	123	41	62	359	216	398	265

### 4.3 Test methods for the properties of the concrete

#### 4.3.1 Compressive strength

**Fig. 4.2:**
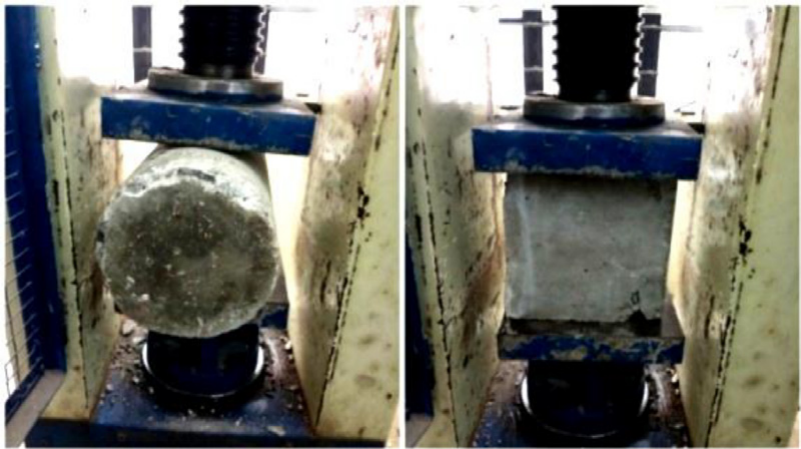
Compressive and Split Tensile Testing of Specimens.

Concrete cubes with dimensions of 150 × 150 × 150 mm were cast, and their curing days were 7, 14, and 28 days, to determine the cube compressive strength of the material according to IS 516 (1959) [16]. Three specimens were tested for each test results. The cube compressive strength (f) was calculated using the basic concept after specimens were dried in the open air and put through Compressive and Split Tensile Testing of Specimens as indicated in Figure 4.2.

F=P/A.

Where, F= Compressive strength (N/mm^2^).

P= Load at failure (N).
A= Area of the specimen (mm^2^).

#### 4.3.2 Split Tensile Strength

Concrete cylinder specimens with a 150 mm diameter and 300 mm length were chosen to test the splitting tensile strength of the material according to IS 5816-1999 [17]. Three specimens were tested for each test results. The specimens were cured for 7, 14, and 28 days of curing before being put through a splitting tensile test using a compressive testing machine. The following formula was used to get the splitting tensile strength (F).

F=2P/πdl (N/mm^2^)

Where, P= Load at failure (N)

d= diameter of specimen (mm)
l= length of specimen (mm)

### 4.4 Outcomes and evaluation

**Fig. 4.3:**
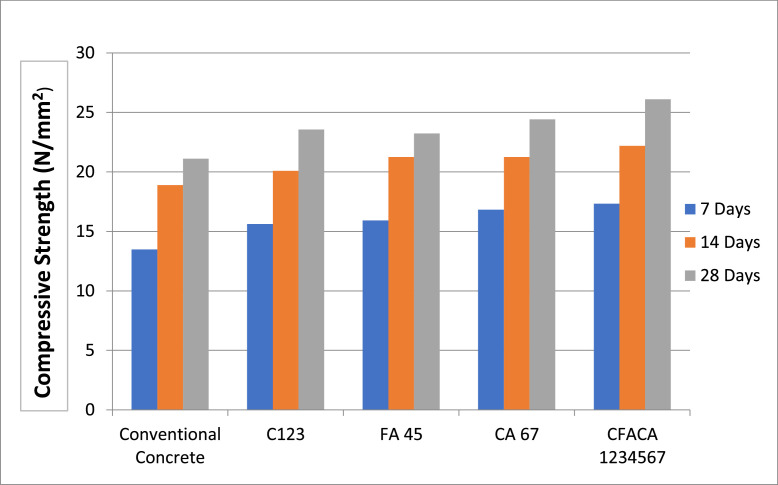
Compressive Strength of the various concrete mixtures studied.

The major problem in the construction field is that there is a scarcity of available materials which are mainly used for preparing concrete. Earlier, for preparing concrete, river sand was used generously, but due to the scarcity of river sand in recent times, in this study, we have used M sand. So, we are replacing the materials of Fine aggregate and Coarse aggregate with the materials that are having the utmost properties of Fine aggregate and Coarse aggregate. So that the replaced concrete will produce the same strength or enhanced strength as the conventional concrete. Properties of hardened concrete are estimated through compressive split tensile strength. Fig. 4.3 shows the Compressive Strength of Conventional specimens and the Concrete Specimens with various supplementary constituents for cement, fine aggregate, and coarse aggregates specimens at age 7, 14 and 28 days respectively. The standard deviation values are presented in Table 6.

**Table 6:** Standard deviation for compressive strength.

Days	Standard deviation				
	Conventional Concrete	C123	FA 45	CA 67	CFACA 1234567
7	0.34	0.46	0.33	0.45	0.54
14	0.21	0.36	0.36	0.47	0.75
28	0.19	0.27	0.41	0.98	0.72

**Fig. 4.4:**
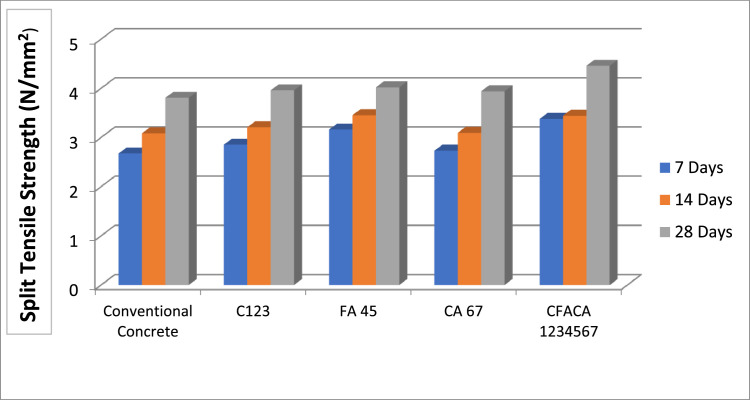
Split tensile Strength analysis of various samples.

Fig. 4.4 shows the Split tensile Strength for Conventional specimens and the Concrete Specimens with various supplementary constituents for cement, fine aggregate and coarse aggregate specimens at age 7, 14 and 28 days respectively. The standard deviation values are presented in Table 7

**Table 7:** Standard deviation for split tensile strength.

Days	Standard deviation				
	Conventional Concrete	C123	FA 45	CA 67	CFACA 1234567
7	0.15	0.10	0.21	0.13	0.05
14	0.13	0.11	0.29	0.03	0.12
28	0.16	0.32	0.11	0.11	0.21

In comparison between conventional specimens and specimens with Cement replaced by Alccofine at 15%, Metakaolin at 10% replacement and GGBBS at 30%, the Percentage of improvement in Compressive Strength is 2.14% and 2.44% at the age of 7 and 28 days, respectively (Fig.4.3 and 4.4). Whereas, the Percentage enhancement in Split Tensile Strength is 0.18% and 0.15% at age 7 and 28 days, respectively.

In assessment amount conventional specimens and the specimens with Fine Aggregate replaced with Copper Slag at a 50% replacement and Foundry Sand at a 30% replacement, the Percentage enhancement in Compressive Strength is 2.43% and 2.12% at age 7 and 28 days, respectively (Fig.4.3 and 4.4). Whereas, at ages 7 and 28 days, the percentage increase in split tensile strength is 0.49% and 0.21%, respectively, respectively. While the comparison between conventional specimens and the Coarse Aggregate replaced with Recycled Aggregates at 20% and Sintered Fly Ash Aggregate at a percentage of 30% specimens, the Percentage Increase in Compressive Strength is 3.35% and 3.30% at age 7 and 28 days, respectively (Fig.4.3 and 4.4). Also, the Percentage improvement in Split Tensile Strength is 0.06% and 0.13% at age 7 and 28 days, respectively.

The Compressive strength and split tensile strength of concrete are increased with the replacement of cement with 15% Alccofine, 10% Metakaolin and 30% GGBFS; the substitute of Fine Aggregate with 50% Copper Slag and 30% Foundry Sand; the replacement of Coarse Aggregate with 20% Recycled Aggregates and 30% Sintered Fly Ash Aggregate, after 28 days of testing as compared to conventional specimens (Fig.4.3 and 4.4). The Percentage enhancement in Compressive Strength is 3.84% and 4.99% at the age of 7 and 28 days, respectively. Whereas, the Percentage Increase in Split Tensile Strength is 0.7% and 0.65% at age 7 and 28 days, respectively.

### 4.5 Summary and Outlook

The materials which we optimized are mostly waste materials obtained from various sources and utilizing it at optimum sources might reduce the cost of construction and high utilization of construction materials without reducing the performance of the structure and thus beneficial for the large scale constructions to produce an eco-friendly structure.

Green cement materials are the future of sustainable construction which do not have negative impact on environment. In this study, an attempt was made to supplement conventional cementitious materials with GGBFS, Metakaolin, Alccofine, the fine aggregate with Copper slag, Foundry sand and coarse aggregate for sintered fly ash aggregate and recycled aggregate.

Compressive and split tensile strength of the materials was enhanced up to 23.63% and 17.06% upon monitoring for 7, 14 and 28 days when the concrete mix was made with the replacement of 30 % GGBFS, 10 % metakaolin and 15 % alccofine for cement, Copper slag and foundry sand as a replacement at a percentage of 50 and 30, Sintered fly ash aggregate and recycled aggregate as an substitute for coarse aggregate at a percentage of optimum 30 and 20 performed better upon comparison with conventional concrete.

## Limitations

Not applicable.

## Ethics Statement

Not applicable.

## CRediT authorship contribution statement

**Nelson Ponnu Durai T.:** Conceptualization, Data curation, Methodology, Investigation, Project administration, Validation, Writing – original draft. **Kandasamy S.:** Supervision, Validation, Writing – review & editing.

## Data Availability

Data set for Publication in Data in brief (Original data) (Mendeley Data).
